# Recovering Ammonia as Ammonium Citrate and Ammonium Sulfate from Sludge Digestion Liquors Using Membrane Contactors in a Pilot Plant

**DOI:** 10.3390/membranes15020062

**Published:** 2025-02-13

**Authors:** Ricardo Reyes Alva, Marius Mohr, Günter E. M. Tovar, Susanne Zibek

**Affiliations:** 1 Institute of Interfacial Process Engineering and Plasma Technology (IGVP), University of Stuttgart, Pfaffenwaldring 31, 70569 Stuttgart, Germany; ricardo.reyes.alva@igb.fraunhofer.de (R.R.A.);; 2 Fraunhofer Institute for Interfacial Engineering and Biotechnology (IGB), Nobelstr. 12, 70569 Stuttgart, Germany

**Keywords:** transmembrane chemical absorption, TMCS, LLMC, membrane stripping, hydrophobic membrane contactor, ammonia recovery, digestate liquor, reject water, trapping solution

## Abstract

Membrane contactors have proved to be effective for recovering ammonia from wastewater by absorbing it into a trapping solution. This study compares the performance of sulfuric acid and citric acid as trapping solutions in a pilot-scale plant for recovering ammonia from sludge digestion liquors using membrane contactors in a liquid–liquid configuration operating at pH 10 and a temperature of 37 °C and using ultrafiltration (UF) technology as pretreatment. The performance of the process using sulfuric acid at a lower pH (9.5) and temperature (30 °C) was also studied, as well as the advantage of including a CO_2_-stripping module in the process. The ammonia elimination efficiency was 88% and 86% when using sulfuric acid and citric acid, respectively. The nitrogen concentration of the produced ammonium sulfate and ammonium citrate reached 23.2 and 14.7 g NH_3_-N·L^−1^, respectively. The ammonia elimination efficiency when using sulfuric acid decreased to 49% when decreasing the pH to 9.5 and to 85% when decreasing the temperature to 31 °C. UF technology was able to reduce the concentration of suspended solids by 90% and the chemical oxygen demand by 37%. However, the UF membranes for the pretreatment and the membrane contactors for ammonia recovery had to be constantly cleaned with acid due to scaling, which significantly increased maintenance efforts. The CO_2_-stripping module reduced the consumption of the caustic soda solution by 23% for increasing the pH level of the treated water. Finally, the specific energy consumption of the plant was 8 kWh·m^−3^.

## 1. Introduction

Ammonium (${NH}_{4}^{+}$) is present in high concentrations (>500 mg·L^−1^) in multiple types of wastewaters, including the side streams produced in municipal wastewater treatment plants (WWTPs) [1]. This important source of nitrogen is required in several anthropogenic activities, including agriculture, and is of such great economic relevance that in 2019 around 235 million tons of ammonia were produced [2]. Furthermore, the synthesis of ammonia via the well-known Haber–Bosch Process demands about 1–2% of the global energy consumption [3]. Still, the objective in conventional WWTPs is not to recover this valuable resource but to remove it via the activated sludge process, which accounts for around 80% of the total energy demand in the treatment plants [4]. Current trends, promoted by sustainable economic models, are allowing the transition of conventional WWTPs towards wastewater biorefineries, in which nitrogen, among other resources, will be recovered from ammonia-rich side streams such as the reject water after sludge anaerobic digestion [5].

In most WWTPs, the anaerobic digestion liquor is recycled back to the activated sludge process, increasing the nitrogen load in the nitrification/denitrification basins. As a result, the nitrous oxide emissions also increase, along with the amount of valuable nitrogen lost as N_2_. From a different perspective, transforming these WWTPs into wastewater biorefineries would enable the recycling-oriented use of nitrogen while also reducing emissions to the atmosphere.

**Figure 1:**
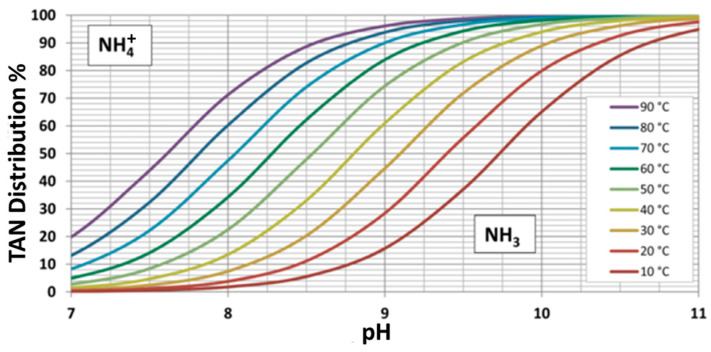
Total ammoniacal nitrogen distribution in the functions of pH and temperature. Reprinted/adapted with permission from [7].

Hydrophobic gas-permeable membranes are among the current technologies available that can be used for recovering nitrogen from wastewater. This type of membrane allows the permeation of gases while retaining liquid substances. This unique feature enables the selective recovery of gaseous ammonia by absorbing it into a trapping solution (TS) flowing on the other side of the membrane through a process called transmembrane chemical absorption (TMCA), or membrane stripping, and erroneously known as transmembrane chemisorption (TMCS) [6]. One prerequisite for increasing the performance of this recovery process is to shift the chemical equilibrium of the total ammoniacal nitrogen (TAN) towards its non-ionic gaseous form (NH_3_), and this can be achieved by increasing pH and temperature, as shown in Figure 1.

The first trials of TMCA for recovering ammonia date to the 1980s [8], and even though nowadays there are already existing full-scale facilities [9,10,11], the process is still facing challenges for improving its economics and increasing its expansion [6]. The major problems identified by multiple authors include the low concentration of nitrogen in the obtained products, the negative process economics due to the intensive use of chemicals and thermal energy for the conditioning of the water, and the pretreatment requirements for reducing membrane fouling [12]. While there is a good amount of literature on the process efficiency at the lab scale when treating synthetic solutions, there are noticeably fewer records on larger-scale experiences treating real wastewater and combining technologies, such as CO_2_ stripping for reducing chemical consumption [6].

Moreover, the available literature is predominantly focused on the process operated with sulfuric acid as the trapping solution [6]. While the obtained ammonium sulfate is a well-established fertilizer, its market value is relatively low (0.16 USD·kg^−1^; November 2024, Europe) [13] and its use in agriculture is mainly limited to sulfur-deficient fields [14]. Increasing the market versatility of the process by producing different types of products according to specific needs could also contribute to its expansion [15]. Thus, it is also important to increase the overall knowledge of the process using alternative capture solutions.

This study aims to contribute to the available literature on the process efficiency at the pilot scale and when coupling it with other technologies. Moreover, this research gives continuity to a previous research paper using citric acid as alternative trapping solution, which, to date, is the only study on TMCA in a liquid–liquid membrane contactor configuration, and it was limited to laboratory conditions [16].

In addition, there is a need to improve the pretreatment of the water before it flows through membrane contactors, especially when using hollow fiber membrane contactors (HFMCs), as they can be easily clogged by solid matter. Regardless of the type of membrane contactors, this technology can also suffer from wetting, leading to the loss of hydrophobic properties [6,12]. The pretreatment should be tailored to the type of wastewater to be treated, and the limited experience at pilot-scale and large-scale TMCA facilities treating reject water from sludge anaerobic digestion has shown the importance of improving this first conditioning step [6,17]. Under this context, it is worth noting that only a few lab-scale studies have combined ultrafiltration (UF) technology with TMCA [18,19]. Even though UF technology can be energy-intensive and requires regular cleaning, it was included in this study as a pretreatment, with the hypothesis that the quality of the permeate produced could be more adequate than that obtained from the treatment train carried out in the aforementioned large-scale facilities.

In summary, this study is the first one reporting the performance of the TMCA process at the pilot scale combining ultrafiltration and CO_2_-stripping modules as pretreatments and citric acid as the trapping solution, thus producing ammonium citrate. Furthermore, this research contributes to the data bank on this process outside of laboratory conditions, including energy and chemical requirements with and without CO_2_ stripping, and thus complements the limited research that has been conducted at the pilot or full scales.

## 2. Materials and Methods

### 2.1. Water Source and Pilot Plant Pretreatment

The pilot plant was installed within the municipal wastewater treatment plant in Erbach (Baden-Württemberg), Germany, with a design capacity of 25,000 population equivalent. In this plant, the anaerobic digestion liquors, also known as reject water, process water, centrate, filtrate or supernatant, are obtained from the dewatering stage of the digestate, which is carried out with a chamber filter press with an absolute rating of 150 µm. The production of this side stream is 35 m^3^·d^−1^ on average and has a TAN concentration of approximately 700 mg·L^−1^ [20]. Thus, the amount of nitrogen that can be potentially recovered in this WWTP through the TMCA process is around 24.5 kg NH_3_-N·d^−1^ or 8940 kg NH_3_-N per year. The pilot plant was designed to treat approximately half of the produced centrate. During regular operation, the digestion liquors are returned to the activated sludge process of the WWTP.

**Figure 2:**
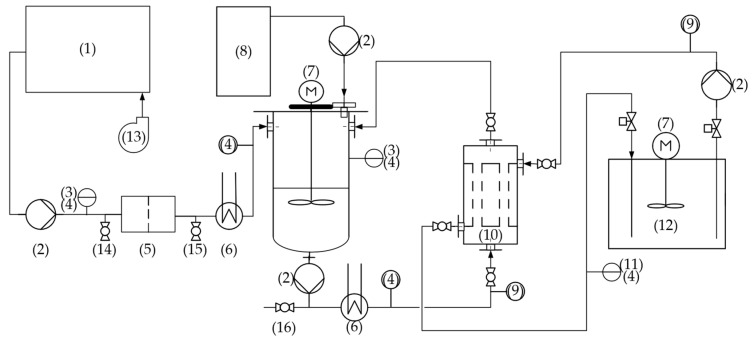
Pilot plant configuration. (1) Feed water reservoir with fine-bubble diffusors; (2) pumps; (3) pH sensor; (4) temperature sensor; (5) filtration module; (6) heat exchangers; (7) agitators; (8) caustic soda container with pallet scale; (9) flowmeters; (10) membrane contactors; (11) electrical conductivity sensor; (12) sulfuric acid container with level sensor; (13) side channel blower; (14) sampling point before UF; (15) sampling point after UF; (16) discharge and sampling point after TMCA.

As depicted in Figure 2, the reject water was first collected in a 5 m^3^ tank that served as a reservoir in which air was blown from the bottom through D-Rex fine-bubble diffusers (OTT Group, Langenhagen, Germany), functioning as the CO_2_-stripping module. Following the recommendations from García-González et al. [21], this process was operated with low aeration rates (6–12 m^−3^·h^−1^ Air·m^−3^ H_2_O) to reduce the amount of ammonia losses due to volatilization and to reduce the risk of possible nitrification. To reduce the content of solids, the water was passed through a cartridge filter with a 50 µm nominal pore size (Wolftechnik, Weil der Stadt, Germany) and downstream through a PVDF cross-flow ultrafiltration membrane (Memos, Pfullingen, Germany) with a molecular weight cut-off of 100 kDa. The temperature of the obtained permeate was increased with the use of cross-flow heat exchangers (FTC, Mainz, Germany).

### 2.2. Reagents and Hydrophobic Gas-Permeable Membrane

Technical sulfuric acid (H_2_SO_4_, CAS 7664-93-9, 50%) and citric acid (C_6_H_8_O_7_, 77-92-9, 50%) solutions were used as ammonia trapping agents. Additional to the CO_2_-stripping module, a sodium hydroxide solution was dosed for increasing the pH of the feed water (NaOH, CAS 1310-73-2, 30%).

The ammonia recovery module consisted of four cased hollow fiber membrane modules (3M™ Liqui-Cel™ EXF-8 × 20) made of polypropylene. Table 1 summarizes the features relevant to this study for a single membrane contactor module [22].

### 2.3. Pilot Plant and Experimental Stages

As recommended by the supplier, the HFMCs were mounted vertically for enabling a complete draining of the system supported with air supply [22]. The configuration was set as a closed-loop circuit, in which the feed water flow was from bottom to top through the shell side of the module, while the acidic trapping solution flowed countercurrent, through the lumen side of the fiber membranes. The four membrane modules were installed in parallel configuration.

After conditioning to the desired pH and temperature, each batch of centrate was recirculated through the membranes at an average flow rate of 6000 L·h^−1^ (approximately 1500 L·h^−1^ per membrane module) for 10 min, while the acid was recirculated at a flow rate four times lower. For all streams of the pilot plant, a composite sample was taken daily, which consisted of a mixture of four equally sized grab samples taken at an interval of one to two hours. The composite samples were analyzed on the same day or refrigerated and analyzed after no more than three days. The container with the TS included a level sensor (Aricon, Solingen, Germany) that allowed to analyze the increase in volume due to the water vapor transport. The trapping solution was continuously stirred to ensure a constant concentration throughout the system. Ceramic pumps (NEES, Walzbachtal, Germany) were used for safely pumping both streams throughout their respective circuits. A peristaltic pump (AxFlow, Düsseldorf, Germany) was used for dosing the NaOH required for increasing and maintaining the pH to the desired levels. The tank containing the NaOH solution was placed over a pallet scale KFN-TAM (KERN & SOHN, Albstadt, Germany) for measuring the caustic soda consumption. All components were connected to power consumption meters from which the energy consumption was obtained. The filtration and CO_2-_stripping modules were connected to one power meter and all other components were connected to another.

Throughout the 5 months of operation, the plant was tested under different operational conditions and settings to study their effect on the performance of the TMCA process. The modified parameters were the pH and temperature of the feed water. A summary of the different experimental stages can be seen in Table 2.

The first stage of experiments served as a benchmark, where the water was conditioned to a pH value of 10 solely with the addition of caustic soda, and to a temperature of 37 °C. The trapping solution used was sulfuric acid as it is the most widely used for this process.

The pretreatment and conditioning in the next stage of operation was identical except for the inclusion of the CO_2_-stripping module for increasing the pH of the feed water, and thus decreasing the NaOH consumption.

The effect of pH and temperature was studied in the subsequent stages by operating at pH 9.5 while keeping the temperature at 37 °C, and then operating the system at a temperature of 31 °C while keeping the pH at 10.

Finally, the system was operated at pH 10 and a temperature of 37 °C using citric acid as the TS instead of sulfuric acid, obtaining ammonium citrate as the product.

### 2.4. Analytical Methods

The measured parameters for the feed water were pH, temperature, electrical conductivity, total ammoniacal nitrogen, total (TA) and phenolphthalein alkalinity (PA), total suspended solids (TSSs), chemical oxygen demand (COD), and reactive orthophosphate (${PO}_{4}^{3-}$-P). For the treated water and acidic solution, only the pH, temperature, electrical conductivity, and ammoniacal nitrogen were measured, and total and carbonate alkalinity were additionally measured for the treated water.

The pH value and temperature were measured with a pH sensor InPro4010/120/PT1000 (Mettler Toledo, Greifensee, Switzerland). The electrical conductivity was measured with a portable WTW-Multi 3320 multimeter (Xylem, Atlanta, GA, USA). TAN, COD, and ${PO}_{4}^{3-}$-P were measured following the persulfate digestion, salicylate, dichromate, and vanadate–molybdate methods, respectively, and with a DR3900 spectrophotometer (Hach, Ames, IA, USA). An important remark is that the acidic samples must be diluted and/or neutralized for the proper measuring of TAN when applying the previously described analytical method. The total suspended solids were measured by gravimetric determination according to the European Standard EN 872:2005 [23]. TA and carbonate alkalinity were measured using AL-AP test kits (Hach, Ames, IA, USA). The water vapor transport was calculated from the volume increase in the tank holding the acidic trapping solution. Finally, the consumption of sodium hydroxide was measured using a KFN-TAM pallet scale (KERN & SOHN, Albstadt, Germany).

### 2.5. Data Analysis

The statistical analysis of data included mean and standard deviation values. Removal efficiency was calculated with Equation (1).

(1) $$\;y_{NH3,rem.\;}(\%)=1-\left(\frac{C_{NH3\;F,f}(\mathrm{mg}\cdot L^{-1})}{C_{NH3\;F,o}(\mathrm{mg}\cdot L^{-1})}\right)\times 100$$

where the *C* stands for concentration while the subscripts *rem*, *F*, *o*, and *f* refer to “removed”, “feed”, “initial”, and “final”, respectively.

The recovered ammonia mass transfer *J_NH3, TS_* was obtained from Equation (2), according to Xu and He [24] and Reig et al. [25].

(2) $$J_{NH3,\;TS}\left({g\cdot m}^{-2}\cdot h^{-1}\right)=\frac{c_{NH3\;TS,f}\left({g\cdot L}^{-1}\right)\cdot V_{TS\;}\left(L\right)}{A_{m}\;(m^{2})\cdot t\left(h\right)}$$

where *A_m_* and the subscript *TS* stand for the total membrane superficial area (212 m^2^) and for the “trapping solution”, respectively. Finally, Equation (3) was used for calculating the water vapor transport (*j_w_*) across the hydrophobic membranes.

(3) $$j_{w}\left(L\cdot m^{-2}\cdot h^{-1}\right)=\frac{V_{w}\left(L\right)}{A_{m}\left(m^{2}\right)\cdot t\left(h\right)}$$

where *V_W_* stands for the volume of water transported from the feed water side into the trapping solution tank.

## 3. Results and Discussion

### 3.1. Pretreatment and Feed Water Characterization

The centrate was analyzed before and after ultrafiltration and after the membrane stripping process as shown in Figure 2. The filtration pretreatment had the main purpose of reducing the risk of clogging and fouling in the hollow fiber membranes downstream. Another important effect of the pretreatment was the reduction in organic compound content. The average specific energy consumption of this module, depicted in Figure 2 as components (1–5) and (13), was 4 kWh per cubic meter of produced permeate. Table 3 summarizes the average characteristics of the process water throughout the entire period of operation.

The electrical conductivity remained almost unaltered after ultrafiltration at around 4.7–4.3 mS·cm^−1^ and it was doubled after the TMCA process due to the added OH- ions from the caustic soda. Similarly, the amount of TDS after the TMCA process increased by a similar proportion to the conductivity, indicating a higher amount of ions in the solution. The total alkalinity of the raw reject water fell considerably below the usual range (2000–4000 mg CaCO_3_·L^−1^) for anaerobic digester liquors in domestic wastewater treatment plants, and this could be explained by the fact that the first sampling point was located downstream from the CO_2_-stripping module, in which alkalinity is consumed [26]. Moreover, the TA also increased with the added NaOH by around 20 percent from the initial value.

The ammoniacal nitrogen concentration of the raw centrate is within the typical range for this type of wastewater coming from municipal WWTPs (>500 mg·L^−1^) and it was slightly reduced after ultrafiltration, possibly due to cake filtration or NH_3_ volatilization [27]. Considering the alkalinity relationships, and the fact that the PA for the raw liquors equals zero, it can be concluded that its TA is bicarbonate alkalinity; thus, its mean concentration can be converted to a value of 17.5 mmol ${HCO}_{3}^{-}$·L^−1^. Assuming that the TAN is present in ionized form, the molar concentration results in 40.3 mmol ${NH}_{4}^{+}$·L^−1^, producing an ${NH}_{4}^{+}/{HCO}_{3}^{-}$ molar ratio of 2.3. Even though Daguerre-Martini et al. [28] stated that the combination of the TMCA process with a CO_2_-stripping module is most adequate when the feed water presents a molar ratio of ${NH}_{4}^{+}/{HCO}_{3}^{-}$ lower than 1, it is pertinent to compare the NaOH consumption with and without the stripping module in place.

The concentration of organic compounds (measured as COD) in the permeate decreased to around 60% with respect to the raw reject water. This effect is highly beneficial for the TMCA process, since according to the literature, organic compounds are the main fouling source when using HFMCs [29].

As mentioned before, HFMCs are prone to becoming clogged with solid compounds, which is why it is of great importance to reduce the content of suspended solids. The UF system achieved a TSS content reduction of over 90%. The concentration of suspended matter in the permeate is considerably below 100 mg·L^−1^, which is the recommendation of Nättorp and Egli [11] as a requirement from their experience operating the same type of membrane in a well-functioning full-scale facility in Switzerland. In addition, the risk of membrane clogging was reduced by flowing the feed water through the shell side of the membrane, which is less sensitive to suspended compounds than the lumen side.

Furthermore, the content of reactive phosphorus was measured to assess the clogging risk of the membranes due to the precipitation of P-salts when conditioning the water to pH > 9. Similar to the case of TAN, phosphate was filtered to some extent despite having a molecular weight much smaller than the membrane’s nominal cut-off capacity. The UF membrane was able to retain around half of the initial orthophosphate presumably through a combination of precipitation and cake filtration, which in this case was also convenient for the stability of the TMCA process.

#### Clean-in-Place (CIP)

Regardless of the relatively “clean” permeate, constant cleaning of the hydrophobic gas permeable membranes was required due to apparent membrane clogging, which was noticeable through a steady decrease in flow rate (<1000 L·h^−1^) through the membrane contactors and a simultaneous pressure increase (>1 bar) in the system. Considering that the suspended solids and the phosphate content were relatively low, the constant clogging of the membrane contactors was presumably produced by calcium carbonate precipitation. The latter hypothesis is backed up by the alkalinity concentration of the feed and treated water, as well as the fact that the drinking water in the region falls within the category of hard water with an average value of 19.6°dh, which is equivalent to 349.8 ppm [30]. The fact that acidic cleaning of the membranes with citric acid was efficient at unclogging the system is another hint that the membranes were suffering from scaling.

The CIP procedure performed was based on the guidelines provided by the membrane manufacturer, in which the recommendations are slightly different depending on the severeness of the fouling. The cleaning procedure described in Table 4 for moderate fouling was sufficient for the operational period of this pilot plant.

The flow rate of the cleaning agents was always within 1–2 m^3^·h^−1^ per membrane contactor. The complete procedure was carried out once per month. However, acidic cleaning was required multiple times per week due to the constant clogging described earlier in this section.

After cleaning, the flow rate of feed water through the membranes was recovered to 1500 L·h^−1^ and the pressure was always reduced to less than 0.5 bar. However, after a few days of operation, sometimes hours, the system would require cleaning again. At this point, the operation was stopped for performing only the acidic cleaning procedure described in Table 4 which was enough to recover the initial operational conditions.

Although the CIP procedure proved to be efficient, this frequency of cleaning would negatively affect the economics of a full-scale operation and would hinder its expansion. In this context, it appears to be imperative to include measures to reduce water hardness in the early stage of the pretreatment of the TMCA process, such as the ones adopted by Nättorp and Egli [11].

### 3.2. TMCA and Performance of the CO_2_-Stripping Module

#### 3.2.1. NH_3_ Mass Transfer, Energy and NaOH Consumption

**Figure 3:**
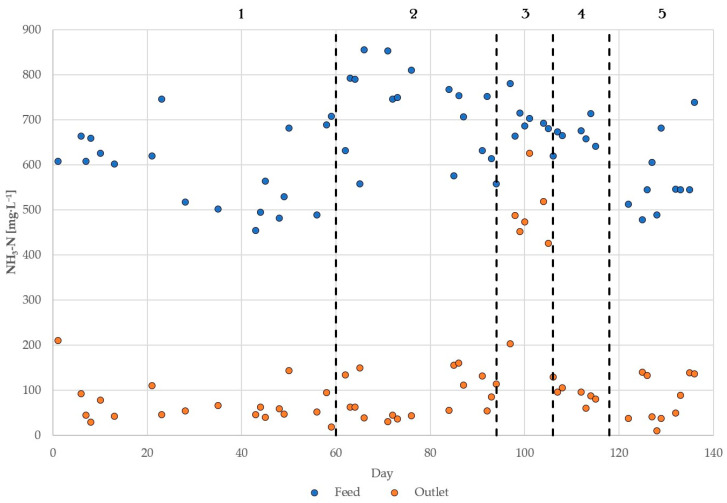
Overview of TAN removal along experimental stages 1–5.

Table 5 shows the mean results of ammonia elimination and mass transfer using Equations (1) and (2) for all experimental stages as well as the consumption of caustic soda solution for conditioning the feed water to pH 10. The ammonia recovery rate provides information on the speed at which ammonia was absorbed in the capture solution. In addition, Figure 3 provides an overview of the feed and outlet’s ammonia concentration during the entire period of operation. The energy consumption of the TMCA process, which is shown in Figure 2 with the exception of components (1–5) and (13), was also 4 kWh per m^3^ of treated water. This means that the pretreatment, including UF and CO_2_ stripping, accounted for 50% of the pilot plant’s energy consumption. In summary, the total energy consumption, including pretreatment, adds up to 8 kWh·m^−3^, which is considerably higher than the 0.5 and 1.5 kWh·m^−3^ reported by Nättorp and Egli [11] for the operation of two full-scale plants treating 133 and 90 m^3^·d^−1^, respectively. The lower energy consumption reported in those plants may be due to the less energy-intensive pretreatment consisting of sand filtration and microfiltration technology, as well as the effect of “energy of scale” produced by the significantly higher throughput. The specific energy consumption of the entire plant can be translated into 16.9 kWh per kilogram of nitrogen recovered, which is twice as high as for the reported green and conventional ammonia synthesis [32]. The specific energy consumption of this plant could be reduced to competitive levels by switching the pretreatment to a less energy-intensive alternative instead of the implemented UF module. Treating a stream with a higher TAN concentration would also improve the specific energy demand, with reject water typically containing 1500 mg·L^−1^ [33].

Naturally, the highest ammonia removal (88%) was obtained during the experimental stage operating at pH 10 and 37 °C, at which, according to Figure 1, the fraction of free ammonia was around 95%. This observation is similar to the one made by Richter et al. [10] when operating a full-scale plant treating 14 m^3^·h^−1^ of reject water from a municipal WWTP at pH 11, where the removal was 85%. Even though the operating pH from that study was higher, the removal efficiency was marginally lower from the one reported in this work, possibly due to a lower operating temperature; however, Richter et al. did not report this parameter. Another operating difference affecting the removal rate of ammonia is the once-through configuration of that system, while in this study, the feed water was recirculated through the membranes as described in the Methodology Section.

When keeping the temperature constant at 37 °C but reducing the pH to 9.5, the N-removal efficiency reduced drastically to 49%. These results are also consistent with the pilot-scale study by Riaño et al. [34], who reported 30–35% of NH_3_-N removal when using digestate produced from swine manure as feed water in a pilot plant operating at pH 8.7–9.1. The operation in both studies involved the recirculation of the process water, but the elimination efficiency was nevertheless low due to the small proportion of gaseous NH_3_ at pH values below 9.5.

Furthermore, only a slight reduction of 2–3 percentage points in TAN removal were noticed when conditioning the centrate to pH 10 and reducing the temperature from 37 °C to 31 °C. Under consideration of this small N removal difference, it is advisable to operate at a lower temperature to reduce the thermal energy requirements of the process. However, this statement should be further studied at pilot- or full-scale facilities and a complete economic analysis should be carried out.

Regarding the rate at which TAN was absorbed in the trapping solution, the highest value was obtained during the first experimental stage, operating at pH 10 and 37 °C and without aeration. When reducing the temperature to 31 °C, the recovery rate decreased just slightly. However, when operating also at benchmark conditions but including the CO_2_-stripping module in the pretreatment, the recovery rate decreased by 17% with respect to the experimental stage without this pretreatment. The reason for this drop in performance could be that the acidic TS was partially loaded with nitrogen in the second experimental stage, while fresh sulfuric acid was used in the first stage. In all experimental stages, the achieved recovery rates were similar to the ones reported (8–14 g·m^−2^·d^−1^) in the pilot-scale study by Riaño et al. [34] treating anaerobic digestion liquors, and the highest nitrogen content achieved in the produced solutions was 2.3%_(*w*/*w*)_. This concentration is significantly lower than that in commercially available ammonium sulfate solutions, which contain around 8% N. In addition, according to the German Fertilizer Ordinance, the nitrogen content of an ammonium sulfate solution produced from treating wastewater must be at least 5% or approximately 50 g N·L^−1^ in order to be classified as a fertilizer [35].

**Figure 4:**
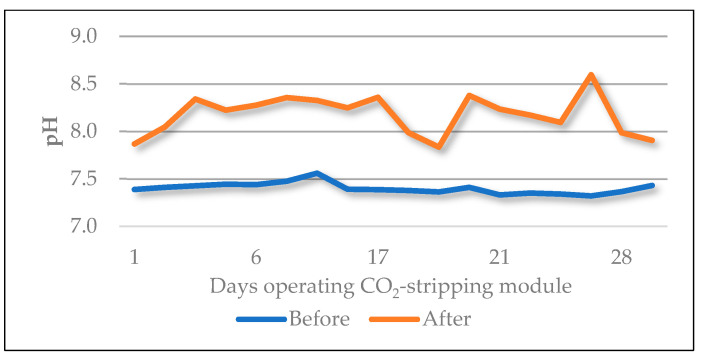
Effect of the CO_2_-stripping module on the pH value.

Finally, it is worth noting that the caustic soda consumption was 23% lower when including the CO_2_-stripping module in the pretreatment train and conditioning the feed water to pH 10. Figure 4 shows how the pH of the feed water was increased without caustic soda addition and solely by aeration. This module achieved an increase in the pH of the feed water of up to 1 unit.

Moreover, the requirement of the NaOH solution was almost 40% lower when reducing the operating pH from 10 to 9.5, and in both cases without the CO_2_ stripping step. The alkali requirements of around 8.3 L·m^−3^ when conditioning to pH 10 without aeration are in line with a previous study performed by this group at the lab scale (8.0 L·m^−3^) [16]. Further studies, such as one conducted by Noriega-Hevia et al. [36], are required to find the optimal feed conditioning that minimizes chemical consumption while maximizing process efficiency and the profitability of the process.

#### 3.2.2. Water Vapor Transport

**Figure 5:**
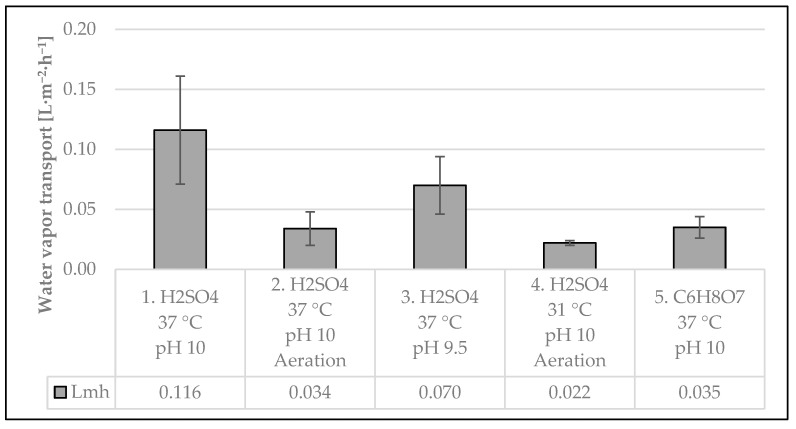
Water vapor transport under different experimental conditions.

It is well known that one of the main challenges faced by the TMCA process is the water vapor transport (WVT) along the membranes, which dilutes the obtained product and increases the consumption of acidic trapping solutions. This phenomena is caused by membrane permeability and the difference in water partial pressure between the process water side and acid side [6]. Figure 5 summarizes the results for WVT, using Equation (3), during the five experimental stages.

Clearly, the highest value of WVT occurred when operating at benchmark conditions with sulfuric acid, which translates to 25 L·h^−1^ when considering a total membrane area of 212 m^2^. When operating the lab-scale module at 40 °C and the same pH and TS, the observed value by this group (0.16 L·m^−2^·h^−1^) when treating the same type of water was 38% higher [16]. Surprisingly, during experimental stage 2, which differs from stage 1 only from the inclusion of the CO_2_-stripping module in the pretreatment, the WVT was 70% lower. To the best of the author’s knowledge, there are no other studies to compare with that evaluated the WVT when operating with and without an aeration module.

The WVT during experimental stage 3 was the second-largest, with 40% less than in stage 1, operating at the same temperature but reducing the pH to 9.5 and also without aeration. This observation is similar in principle but differs in magnitude from the results obtained at the lab scale, in which, by keeping the temperature constant at 40 °C and decreasing the pH from 10.5 to 10, the WVT also decreased, but only by 6%.

As expected, the lowest water vapor transport through the membranes was observed during experimental stage 4, in which the operational temperature (31 °C) was also the lowest. The result under these conditions was 81% lower than when operating at the same pH but at 37 °C, and without aeration. In contrast, the WVT observed during stage 4 is only 35% lower compared to stage 2, which, as mentioned before, is the same as stage 1 but without aeration included. Based on these results, there appears to be a correlation between aeration and the reduction in water vapor transport during the TMCA process. This effect should be further studied at pilot- and full-scale facilities to confirm the abovementioned apparent causal relationship.

Finally, the WVT observed during stage 5, operating at benchmark conditions without aeration but using citric acid as the trapping solution, was 70% lower than when operating with the exact same configuration but using sulfuric acid as the TS. This observation is similar to one made in the lab-scale study, where, when operating at pH 10 and 40 °C, the WVT when using citric acid was 83% lower than when using sulfuric acid [16]. However, the water vapor transport when using citric acid without aeration was practically the same as when using sulfuric acid with aeration under the same pH and temperature conditions.

## 4. Conclusions

This study has demonstrated on a pilot scale that citric acid can effectively serve as a trapping solution in the transmembrane chemical absorption process for the recovery of ammonia as ammonium citrate from sludge digestion liquors. The performance of citric acid as a TS is lower than that of sulfuric acid in terms of ammonia recovery efficiency, but higher in terms of reduced water vapor transport when aeration for CO_2_ stripping was not included in both cases. Moreover, the WVT observed during operation with sulfuric acid was 70% lower with the stripping module than without stripping. It would be necessary to carry out further studies at pilot and large scales to verify the influence of aeration on water vapor transport. The use of an ultrafiltration membrane module as a pretreatment required regular maintenance and accounted for 50% (including the aeration module) of the total energy consumption of the process, but was highly effective in reducing the suspended solids (>90%) and organic matter content (37%). However, further measures must be taken in the pretreatment to reduce the concentration of precipitable dissolved solids in order to protect the membrane contactors and reduce the frequency of their cleaning. The specific energy consumption of the plant was considerably higher than that of similar plants and of the process for ammonia synthesis. Implementing a less energy-intensive pretreatment is important for improving the competitiveness of the process. Finally, including a CO_2_-stripping module in the pretreatment can reduce the requirements of caustic soda by more than 20%. In order to further expand and develop the TMCA process, it is essential to continue pilot- and full-scale studies to improve the efficiency and cost of the pretreatment. Further studies are also needed to increase product concentration, which in this study was not higher than 23.2 g NH_3_-N·L^−1^.

## Figures and Tables

**Table 1:** Membrane contactor characteristics.

Feature	Unit	Value
Membrane type	-	Hollow fibers X-50
Material	-	Polypropylene
Effective surface area	m^2^	53
Area density	m^2^·m^−3^	1871
Number of fibers	-	156,000
Pore size	µm	0.04

**Table 2:** Description of the operational stages.

Stage	Pretreatment	CO_2_ Stripping	pH	T [°C]	Acid
1	UF Module	No	10	37	Sulfuric
2	UF Module	Yes	10	37	Sulfuric
3	UF Module	No	9.5	37	Sulfuric
4	UF Module	Yes	10	31	Sulfuric
5	UF Module	No	10	37	Citric

**Table 3:** Characteristics of the treated centrate water. Standard deviation values are given in parentheses.

Parameter	Units	Before UF	After UF	After TMCA
Electrical conductivity (σ)	mS·cm^−1^	4.7 (0.6)	4.3 (0.5)	8.7(2.9) *
Total alkalinity (TA)	mg CaCO_3_·L^−1^	875.5 (214.9)	768 (139.1)	928.5 (189.5)
Phenolphthalein alkalinity (PA)	mg CaCO_3_·L^−1^	0 (0)	0 (0)	719.7 (194.4)
Total suspended solids (TSS)	mg·L^−1^	254.4 (175.8)	25.1 (14.1)	**
Total dissolved solids (TDS)	mg·L^−1^	5.3 (0.4)	4.8 (0.5)	10.2 (4.4) *
Total ammoniacal nitrogen (TAN)	mg·L^−1^	725.1 (161.8)	647.9 (104.3)	126.7 (135.1) *
Chemical oxygen demand (COD)	mg·L^−1^	206.3 (43.2)	130.8 (28.3)	**
Orthophosphate (PO_4_-P)	mg·L^−1^	12.4 (2.5)	6.0 (2.7)	**

* The standard deviation is large because the value depends greatly on the experimental stage. ** = Not measured.

**Table 4:** Cleaning procedure against moderate fouling for Liqui-Cel™ membranes [31].

Step	Parameter	Duration
1	Flushing with demin. water	10 min
2	Cleaning with 6%*_v_*_/*v*_ NaOH solution	30–60 min
3	Drain system	
4	Cleaning with 10%*_v_*_/*v*_ citric acid solution	30–60 min
5	Drain system	
6	Flushing with demin. water	10 min
7	Drain system	

**Table 5:** Ammonia mass transfer and caustic soda consumption under the studied conditions. Standard deviation values are given in parentheses.

Experimental Stage	TAN*_o_* [mg·L^−1^]	TAN*_f_* [mg·L^−1^]	Removal [%]	TAN*_TS_* [mg·L^−1^]	Vol.*_TS_* [L]	TAN Recovery Rate [g·m^−2^·d^−1^]	NaOH 30% Consumption [L·m^−3^]
1	563.8 (96.4)	65.3 (29.8)	88	12,100	1624	15.9	8.3 (2.3)
2	730.5 (90.6)	92.7 (49.6)	87	16,640	2581	13.2	6.4 (1.1)
3	686.3 (67.8)	348.5 (221.7)	49	*	*	*	5.1 (1.0)
4	659.6 (29.7)	98.4 (23.8)	85	23,150	1294	13.5	6.6 (0.7)
5	583 (92.7)	81.3 (51.6)	86	14,700	1717	15.7	6.4 (0.8)

*o* = initial; *f* = final; *TS* = ammonium trapping solution; * = operational period was too short.

## Data Availability

The data presented in this study are available on request from the corresponding author.

## References

[1] Licon Bernal E.E., Maya C., Valderrama C., Cortina J. (2016). Valorization of ammonia concentrates from treated urban wastewater using liquid–liquid membrane contactors. Chem. Eng. J..

[2] Ghavam S., Vahdati M., Wilson I.A.G., Styring P. (2021). Sustainable Ammonia Production Processes. Front. Energy Res..

[3] Guillen-Burrieza E., Moritz E., Hobisch M., Muster-Slawitsch B. (2023). Recovery of ammonia from centrate water in urban waste water treatment plants via direct contact membrane distillation: Process performance in long-term pilot-scale operation. J. Membr. Sci..

[4] Dow N., Saldin T.F., Duke M., Yang X. (2022). Pilot demonstration of nitrogen removal from municipal wastewater by vacuum membrane distillation. J. Water Process Eng..

[5] Darestani M., Haigh V., Couperthwaite S.J., Millar G.J., Nghiem L.D. (2017). Hollow fibre membrane contactors for ammonia recovery: Current status and future developments. J. Environ. Chem. Eng..

[6] Gonzalez-Salgado I., Guigui C., Sperandio M. (2022). Transmembrane chemical absorption technology for ammonia recovery from wastewater: A critical review. Chem. Eng. J..

[7] PONDUS Verfahrenstechnik GmbH Stickstoffrückgewinnung aus Schlämmen und Abwässern: Das Ammonium-Ammoniak-Gleichgewicht. https://www.pondus-verfahren.de/.

[8] Imai M., Furusaki S., Miyauchi T. (1982). Separation of volatile materials by gas membranes. Ind. Eng. Chem. Proc. Des. Dev..

[9] Ulbricht M., Schneider J., Stasiak M., Sengupta A. (2013). Ammonia Recovery from Industrial Wastewater by TransMembraneChemiSorption. Chem. Ing. Tech..

[10] Richter L., Paulsen S., Wichern M., Grömping M., Robecke U., Haberkamp J. (2021). Hollow-fibre Membrane Contactors for Nitrogen Recovery from Municipal Wastewater. Chem. Ing. Tech..

[11] Nättorp A., Egli C. New Approaches and Best Practices for Closing the Materials Cycle in the Water Sector: Ammonium Sulphate Production via Membrane Stripping in Altenrhein (CH), Water Europe, 2023. https://mp.watereurope.eu/d/technology/1112/.

[12] Jang Y., Lee W., Park J., Choi Y. (2022). Recovery of ammonia from wastewater by liquid–liquid membrane contactor A review. Membr. Water Treat..

[13] BUSINESS analytiQ Ammonium Sulfate Price Index. https://businessanalytiq.com/procurementanalytics/index/ammonium-sulfate-index/.

[14] Soto-Herranz M., Sánchez-Báscones M., Antolín-Rodríguez J.M., Martín-Ramos P. (2022). Evaluation of Different Capture Solutions for Ammonia Recovery in Suspended Gas Permeable Membrane Systems. Membranes.

[15] Vecino X., Reig M., Bhushan B., Gibert O., Valderrama C., Cortina J.L. (2019). Liquid fertilizer production by ammonia recovery from treated ammonia-rich regenerated streams using liquid-liquid membrane contactors. Chem. Eng. J..

[16] Reyes Alva R., Mohr M., Zibek S. (2024). Transmembrane Chemical Absorption Process for Recovering Ammonia as an Organic Fertilizer Using Citric Acid as the Trapping Solution. Membranes.

[17] Richter L., Wichern M., Grömping M., Robecke U., Haberkamp J. (2020). Ammonium recovery from process water of digested sludge dewatering by membrane contactors. Water Pract. Technol..

[18] Du Preez J., Norddahl B., Christensen K. (2005). The BIOREK^®^ concept: A hybrid membrane bioreactor concept for very strong wastewater. Desalination.

[19] Wäeger-Baumann F., Fuchs W. (2012). The Application of Membrane Contactors for the Removal of Ammonium from Anaerobic Digester Effluent. Sep. Sci. Technol..

[20] Stadt Erbach (2021). Jahresbericht_PLS_2018-2021_Zusammenstellung.

[21] García-González M.C., Vanotti M.B., Szogi A.A. (2015). Recovery of ammonia from swine manure using gas-permeable membranes: Effect of aeration. J. Environ. Manag..

[22] 3M User Guide: 3M™ Liqui-Cel™ Membrane Contactors for TransMembrane ChemiSorption Operation. https://www.3m.com/3M/en_US/liquicel-us/resources/operating-and-technical-guides/.

[23] (2005). Water Quality—Determination of Suspended Solids: Method by Filtration Through Glass Fibre Filters.

[24] Xu B., He Z. (2021). Ammonia recovery from simulated anaerobic digestate using a two-stage direct contact membrane distillation process. Water Environ. Res..

[25] Reig M., Vecino X., Gibert O., Valderrama C., Cortina J.L. (2021). Study of the operational parameters in the hollow fibre liquid-liquid membrane contactors process for ammonia valorisation as liquid fertiliser. Sep. Purif. Technol..

[26] Alpha Analytical, Inc (2012). Alkalinity, Titration Method.

[27] Deng Z., van Linden N., Guillen E., Spanjers H., van Lier J.B. (2021). Recovery and applications of ammoniacal nitrogen from nitrogen-loaded residual streams: A review. J. Environ. Manag..

[28] Daguerre-Martini S., Vanotti M.B., Rodriguez-Pastor M., Rosal A., Moral R. (2018). Nitrogen recovery from wastewater using gas-permeable membranes: Impact of inorganic carbon content and natural organic matter. Water Res..

[29] Zarebska A., Karring H., Christensen M.L., Hjorth M., Christensen K.V., Norddahl B. (2017). Ammonia Recovery from Pig Slurry Using a Membrane Contactor—Influence of Slurry Pretreatment. Water Air Soil Pollut..

[30] Stadt Erbach Wasserversorgung: Wasserhärte und ph-Werte. https://www.erbach-donau.de/cms/BuergerService-Ver-Entsorgung-Wasserversorgung.html.

[31] 3M 3M™ Liqui-Cel™ EXF Series Membrane Contactors: Cleaning and Storage Guidelines 3M. https://www.3m.com/3M/en_US/liquicel-us/resources/operating-and-technical-guides/.

[32] de la Hera G., Ruiz-Gutiérrez G., Viguri J.R., Galán B. (2024). Flexible Green Ammonia Production Plants: Small-Scale Simulations Based on Energy Aspects. Environments.

[33] Karmann C., Mágrová A., Jeníček P., Bartáček J., Kouba V. (2024). Advances in nitrogen removal and recovery technologies from reject water: Economic and environmental perspectives. Bioresour. Technol..

[34] Riaño B., Molinuevo-Salces B., Vanotti M.B., García-González M.C. (2021). Ammonia Recovery from Digestate Using Gas-Permeable Membranes: A Pilot-Scale Study. Environments.

[35] Federal Ministry of Justice Verordnung Über das Inverkehrbringen von Düngemitteln, Bodenhilfsstoffen, Kultursubstraten und Pflanzenhilfsmitteln 1 (Düngemittelverordnung—DüMV): Definition von Düngemitteltypen. https://www.gesetze-im-internet.de/d_mv_2012/anlage_1.html.

[36] Noriega-Hevia G., Serralta J., Seco A., Ferrer J. (2021). Economic analysis of the scale-up and implantation of a hollow fibre membrane contactor plant for nitrogen recovery in a full-scale wastewater treatment plant. Sep. Purif. Technol..

